# Catabolic and regulatory systems in *Shewanella oneidensis* MR-1 involved in electricity generation in microbial fuel cells

**DOI:** 10.3389/fmicb.2015.00609

**Published:** 2015-06-16

**Authors:** Atsushi Kouzuma, Takuya Kasai, Atsumi Hirose, Kazuya Watanabe

**Affiliations:** School of Life Sciences, Tokyo University of Pharmacy and Life Sciences, Hachioji, Japan

**Keywords:** extracellular electron transfer, bioelectrochemical system, anaerobic respiration, transcriptional regulation, catabolic pathways

## Abstract

*Shewanella oneidensis* MR-1 is a facultative anaerobe that respires using a variety of inorganic and organic compounds. MR-1 is also capable of utilizing extracellular solid materials, including anodes in microbial fuel cells (MFCs), as electron acceptors, thereby enabling electricity generation. As MFCs have the potential to generate electricity from biomass waste and wastewater, MR-1 has been extensively studied to identify the molecular systems that are involved in electricity generation in MFCs. These studies have demonstrated the importance of extracellular electron-transfer (EET) pathways that electrically connect the quinone pool in the cytoplasmic membrane to extracellular electron acceptors. Electricity generation is also dependent on intracellular catabolic pathways that oxidize electron donors, such as lactate, and regulatory systems that control the expression of genes encoding the components of catabolic and electron-transfer pathways. In addition, recent findings suggest that cell-surface polymers, e.g., exopolysaccharides, and secreted chemicals, which function as electron shuttles, are also involved in electricity generation. Despite these advances in our knowledge on the EET processes in MR-1, further efforts are necessary to fully understand the underlying intra- and extracellular molecular systems for electricity generation in MFCs. We suggest that investigating how MR-1 coordinates these systems to efficiently transfer electrons to electrodes and conserve electrochemical energy for cell proliferation is important for establishing the biological basis for MFCs.

## Introduction

Microbial fuel cells (MFCs) are devices that use living microbes as catalysts for the conversion of fuels, such as organic compounds, into electricity (Logan et al., 2006; Watanabe, 2008). In MFCs, electrons released by the oxidative catabolism of organic substrates in bacterial cells are transferred to extracellular electrodes, resulting in electricity generation. The natural diversity of bacterial catabolic activities provides MFCs with a great advantage over chemical fuel cells, which typically require purified reactive fuels, such as hydrogen. MFCs are able to generate electricity from a variety of organic substrates, including sugars (Rabaey et al., 2003), cellulose (Ishii et al., 2008), organic acids (Yates et al., 2012), and wastewater pollutants (Miyahara et al., 2012; Yu et al., 2012).

In most MFCs, bacteria, particularly those affiliated with the phylum *Proteobacteria*, mediate the transfer of electrons to anodes (Logan, 2009). These bacteria possess electron-transfer pathways that electrically connect intracellular oxidative catabolic reactions to extracellular electrodes. Certain species of dissimilatory metal-reducing bacteria (DMRB), such as members of the genus *Shewanella*, intrinsically possess such pathways (termed extracellular electron-transfer (EET) pathways), and are therefore able to use electrodes as terminal electron acceptors for respiration (electrode respiration; Shi et al., 2007). *Shewanella* species belong to the class *Gammaproteobacteria* and are widely distributed in nature, including marine, freshwater, sedimentary, and soil environments (Fredrickson et al., 2008). Members of this genus have attracted considerable recent attention due to their respiratory versatility and potential applicability to biotechnological processes, such as bioremediation (Hau and Gralnick, 2007) and MFCs (Kim et al., 1999).

*Shewanella oneidensis* MR-1 is the most extensively studied strain in the genus *Shewanella* due to its annotated genome sequence (Heidelberg et al., 2002), genetic accessibility, and respiratory versatility (Myers and Nealson, 1988b). This bacterium can respire using a wide variety of organic and inorganic substrates as electron acceptors, including oxygen, fumarate, nitrate, nitrite, thiosulfate, elemental sulfur, trimethylamine N-oxide, dimethyl sulfoxide (DMSO), and anthraquinone-2,6-disulfonate, as well as both soluble and solid metals such as iron, manganese, uranium, chromium, cobalt, technetium, and vanadium (Fredrickson et al., 2008). In addition, MR-1 can transfer electrons to anodes and generate electricity in MFCs without adding exogenous mediators (Kim et al., 1999). For these reasons, MR-1 is a model organism for investigating how bacteria utilize extracellular electron acceptors and generate electricity in MFCs.

The major components of the EET pathway (Mtr pathway) in MR-1 critical for electricity generation in MFCs have been identified. Intracellular catabolic pathways that produce reducing equivalents (e.g., NADH) have also been extensively studied in this species. In addition, several studies have analyzed the transcriptional regulatory systems that control catabolic and electron-transfer pathways in MR-1. In this article, we summarize the current knowledge on catabolic and regulatory systems in *S. oneidensis* MR-1 that are involved in electricity generation in MFCs. The findings from genetic and biochemical studies were reviewed to provide a detailed view of the molecular mechanisms that are directly or indirectly involved in electricity generation by MR-1.

## EET Pathway

**FIGURE 1:**
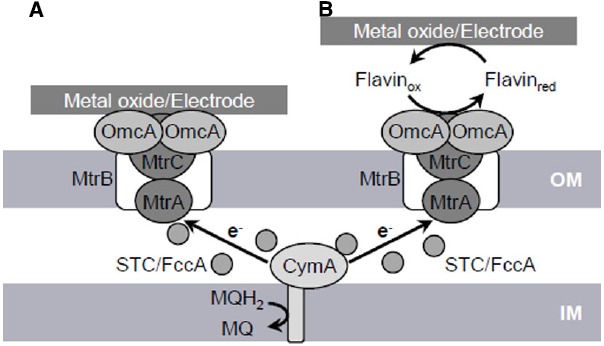
**Proposed extracellular electron transfer (EET) pathways (Mtr pathway) in *S. oneidensis* MR-1 involved in direct EET (A) and mediated EET (B).** OM, outer membrane; IM, inner membrane; MQH_2_, reduced form of menaquinone; MQ, oxidized form of menaquinone.

The respiration of solid metals and electrodes requires a distinct molecular pathway, i.e., the EET pathway, for transferring electrons from intracellular electron carriers (e.g., NADH and quinones), across the inner membrane (IM) and outer membrane (OM), to extracellularly located insoluble electron acceptors. Genetic and biochemical studies have identified five primary protein components, CymA, MtrA, MtrB, MtrC, and OmcA, comprising the EET pathway in *S. oneidensis* MR-1 (the Mtr pathway; Figure 1; Shi et al., 2007). In addition, recent studies have demonstrated that the periplasmic cytochrome pool, which mainly consists of small tetraheme cytochromes (STCs; also referred to as CctA) and flavocytochrome c (FccA) proteins, is also involved in the EET process (Fonseca et al., 2012; Sturm et al., 2015). These findings indicate that the Mtr pathway serves as the major electron conduit that links the IM quinone pool to extracellular solid electron acceptors via a series of electron-transfer reactions between these component proteins.

In the Mtr pathway, EET is initiated by the transfer of electrons from the IM quinone pool to IM-anchored CymA (SO_4591). CymA is a tetraheme c-type cytochrome belonging to the NapC/NirT protein family and consists of a short N-terminal region that is anchored in the IM and a long C-terminal region that protrudes into the periplasm (Myers and Myers, 1997, 2000). The C-terminal region contains four heme-binding sites and mediate electron transfer to a decaheme c-type cytochrome, MtrA, as well as to other periplasmic respiratory proteins, including those responsible for the reduction of DMSO, fumarate, nitrate, and nitrite (Schwalb et al., 2002, 2003; Pitts et al., 2003; Gao et al., 2009; Schuetz et al., 2009).

MtrA (SO_1777) is regarded as a key protein for electron transfer to OM c-type cytochromes (OM-cyts), such as MtrC and OmcA, based on its periplasmic localization and biochemical association with MtrB and MtrC (Hartshorne et al., 2009; Schuetz et al., 2009). MtrB (SO_1776) is an OM-located β-barrel protein consisting of transmembrane β-strands and is required for metal and electrode respiration (Beliaev and Saffarini, 1998; Bretschger et al., 2007). Evidence suggests that MtrB is required for the proper localization and insertion of MtrC and OmcA into the OM (Myers and Myers, 2002). It has also been reported that MtrB forms a stable complex with MtrA and MtrC at a stoichiometry of 1:1:1 and supports electron exchange between these c-type cytochromes by serving as an OM-spanning sheath (Ross et al., 2007; Hartshorne et al., 2009). Interestingly, MtrA was detected in the periplasmic fraction of an *mtrB*-deletion strain, but is localized in the OM fraction in wild-type MR-1, indicating that MtrB supports the *in vivo* localization of MtrA to the OM, although MtrA *per se* is a soluble protein (Hartshorne et al., 2009).

The OM-cyts OmcA (SO_1779) and MtrC (SO_1778) contain 10 heme-binding sites and serve as the terminal reductases for extracellular electron acceptors in the Mtr pathway (Myers and Myers, 2003a, 2004). These OM-cyts are transported to the OM surface by the type-II protein-secretion system (Shi et al., 2008). Biochemical data indicate that OmcA and MtrC form a complex with a stoichiometry of approximately 2:1 (Shi et al., 2006). Genetic studies with MR-1 have demonstrated that current generation in MFCs and reduction rates for insoluble minerals, such as Mn(IV) and Fe(III) oxides, are decreased in single-knockout mutants of *mtrC* and *omcA* and severely impaired in a *mtrC*/*omcA* double-knockout mutant (Beliaev et al., 2001; Myers and Myers, 2001; Bretschger et al., 2007; Newton et al., 2009). In addition, Bretschger et al. (2007) reported that an *mtrC*-overexpressing strain generated 35% more current in an MFC than wild-type MR-1. These observations clearly indicate that OmcA and MtrC play crucial roles in mediating EET reactions at the OM surface. It is likely that the functions of these two OM-cyts partially overlap, as purified MtrC and OmcA proteins are both able to reduce solid Fe(III) oxides (Xiong et al., 2006; Lower et al., 2007), and the overproduction of MtrC can restore the ability of an *omcA*-deletion mutant to reduce MnO_2_ (Myers and Myers, 2003b). However, several lines of evidence indicate that functional differences exist between these two OM-cyts. For instance, Lower et al. (2007) reported that OmcA shows a higher affinity toward hematite (α-Fe_2_O_3_) than MtrC. Furthermore, MtrC appears to play a dominant role in electron-transfer reactions to electrodes, whereas OmcA plays a preferential role in the attachment of cells to solid surfaces (Coursolle et al., 2010; Mitchell et al., 2012).

Although many studies have demonstrated that OmcA and the MtrCAB complex play key roles in the Mtr pathway, it is not yet fully understood how electrons are transferred from IM-anchored CymA across the periplasmic space, which has an average distance of 23.5 nm, to the OM components of this pathway (Dohnalkova et al., 2011). One possible explanation is that soluble electron carrier proteins diffuse through the periplasm and mediate electron transfer between CymA and OM-cyts. Consistent with this hypothesis, Fonseca et al. (2012) reported that soluble periplasmic cytochromes, STC (SO_2727) and FccA (SO_0970), interact with both CymA and MtrA with relatively large dissociation constants and thereby promote transient electron-transfer reactions between CymA and MtrA. Furthermore, Sturm et al. (2015) recently reported that the periplasmic space of MR-1 contains abundant soluble c-type cytochromes (approximately 350,000 hemes per cell), and that a double-deletion mutant of *fccA* and *stc* (*cctA*) exhibits substantial growth deficiencies on ferric iron and other soluble electron acceptors. These observations suggest that the periplasmic cytochrome pool, which mainly consists of STC and FccA, plays important roles in mediating electron transfer from CymA to OM-cyts in the Mtr pathway.

Studies have indicated that electrons are transferred from OM-cyts to electrodes via two pathways, direct electron transfer (DET) and mediated electron transfer (MET) pathways (Baron et al., 2009). In DET, electrons are directly transferred from OM-cyts to solid electron acceptors (Xiong et al., 2006; Lower et al., 2007). In contrast, MET involves the transfer of electrons from OM-cyts to distant solid electron acceptors via secreted electron-shuttle compounds, such as flavins (Marsili et al., 2008; von Canstein et al., 2008). Support for the DET process in MR-1 is based on the fact that purified OmcA and MtrC proteins strongly bind and transfer electrons to crystalline Fe(III) oxides and graphite electrodes (Xiong et al., 2006; Lower et al., 2007). Evidence for MET includes the finding that MR-1 can reduce Fe(III) oxides located at a distance from cells and without direct contact (Lies et al., 2005). In addition to this observation, von Canstein et al. (2008) and Marsili et al. (2008) demonstrated that *Shewanella* spp. secrete flavins, including riboflavin and flavin mononucleotide (FMN), which function as electron shuttles for MET. Notably, however, Coursolle et al. (2010) demonstrated that the majority (∼90%) of electrons transferred to flavins are released from OmcA and MtrC, indicating that these OM-cyts are also required for MET.

Although MR-1 appears to utilize both DET and MET pathways, several lines of evidence indicate that soluble flavins are indispensable for EET under physiological conditions. For instance, Ross et al. (2009) reported that the direct reduction of insoluble metal oxides by OmcA and MtrC proceeds too slowly to explain the physiological rates of electron transfer, and that the reaction rates of these OM-cyts are markedly increased in the presence of flavins. In addition, Marsili et al. (2008) demonstrated that the accumulation of flavins in MR-1 biofilms increased the rate of electron transfer to an electrode by over threefold. Furthermore, Okamoto et al. (2013) reported that one-electron-reduced flavins bind to OM-cyts as redox cofactors, thereby enhancing the rate of electron transfer at the cell/electrode interface. Together, these studies demonstrate that flavins serve crucial functions in EET via the Mtr pathway.

**FIGURE 2:**
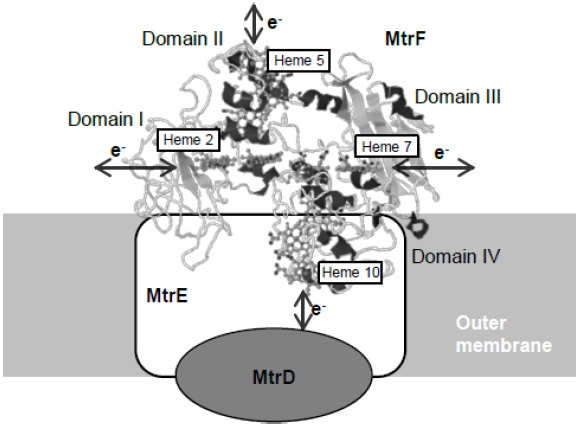
**Crystal structure of MtrF and proposed spatial arrangement at the cell surface.** Predicted sites for electric connections with extracellular substances are indicated with arrows. The structure model of MtrF was obtained from PDB (PDB ID: 3PMQ) and rendered using the Jmol software (http://www.jmol.org/).

The X-ray crystal structures of two OM-cyts, MtrF (MtrC homolog) and OmcA, have been resolved to date (Clarke et al., 2011; Edwards et al., 2014), and have provided insights into how electrons are transferred through these decaheme OM-cyts. Clarke et al. (2011) demonstrated that the 10 hemes in MtrF are arranged at a distance of 7 Å from each other, forming an intramolecular electron conduit with a unique “staggered cross” conformation. Based on the heme arrangement and domain configuration of MtrF, four hemes (hemes 2, 5, 7, and 10 located in domains I, II, III, and IV, respectively) that are potentially important for exchanging electrons with other molecules were identified (Figure 2). Heme 10 is located at the solvent-exposed terminus of the heme chain and is likely involved in receiving electrons from the MtrDE complex (an electron transfer module homologous to the MtrBA complex). In contrast, heme 5, which is located at the opposite end of the protein, is predicted to be responsible for releasing electrons to extracellular electron acceptors. Hemes 2 and 7, which are located in Greek key split β-barrel domains and contain putative FMN-binding sites (domains I and III), are regarded as possible sites for electron exchange with electron shuttles, such as flavins. Edwards et al. (2014) resolved the crystal structure of OmcA, and reported that its heme arrangement and domain configuration are similar to those of MtrF. These researchers also constructed a model structure of MtrC based on the crystal structure of MtrF, and speculated that the electrostatic surface surrounding heme 7 differ between MtrC and MtrF, suggesting that these OM-cyts may differentially interact with substrates (Edwards et al., 2012).

## Transcriptional Regulation of EET-Related Genes

**FIGURE 3:**
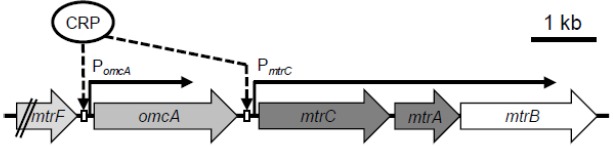
**Organization and proposed transcriptional mechanisms of the *mtr* genes.** Two CRP-binding sites adjacent to promoters of *omcA* (P*_omcA_*) and *mtrC* (P*_mtrC_*) are shown (Kasai et al., 2015).

In contrast to the extensive biochemical characterization of the Mtr pathway, limited studies have examined how MR-1 regulates EET-related genes at the transcriptional level. In the MR-1 genome, the four genes encoding the proteins comprising OM-cyts in the Mtr pathway (*omcA*-*mtrCAB*; the *mtr* genes) are organized in a cluster and oriented in the same direction (Figure 3). Transcriptional analyses of the *mtr* genes have confirmed that *mtrC*, *mtrA*, and *mtrB* are co-transcribed as an operon (Beliaev et al., 2001; Kasai et al., 2015), a finding that is consistent with the biochemical data showing that the *mtr* gene products form a complex (the MtrCAB complex) at 1:1:1 stoichiometry (Ross et al., 2007). In the *mtr* gene cluster, two different transcription start sites (and promoters) have been identified in the upstream regions of *omcA* and *mtrC* (Beliaev et al., 2001; Shao et al., 2014; Kasai et al., 2015), suggesting that *omcA* and *mtrC* are independently regulated. Previous studies have also demonstrated that a cyclic AMP (cAMP) receptor protein (CRP) and adenylate cyclase (CyaC) are essential for the transcriptional activation of the *mtr* genes (Saffarini et al., 2003; Charania et al., 2009). However, upstream signal transduction pathways involved in the cAMP/CRP-dependent transcriptional activation of the *mtr* genes remain to be elucidated.

In *Escherichia coli*, CRP is a well-known global regulator, and functions in conjugation with cAMP, which is an effector molecule of CRP and serves as a signaling molecule for the expression of numerous genes, including those involved in carbon catabolite repression (Botsford and Harman, 1992). When complexed with cAMP, CRP binds to target DNA sequences, resulting principally in the transcriptional activation of downstream genes, such as those involved in sugar metabolism (Botsford and Harman, 1992; Hollands et al., 2007). In *Shewanella* spp., however, evidence suggests that CRP is mainly involved in the regulation of anaerobic respiration (Saffarini et al., 2003; Charania et al., 2009; Murphy et al., 2009). For example, the cAMP/CRP-dependent regulatory system is reported to be essential for regulating anaerobic arsenate reduction in *Shewanella* sp. strain ANA-3 (Murphy et al., 2009), and studies on MR-1 have revealed that CRP is required for the transcriptional activation of genes involved in the reduction of many electron acceptors, including metal oxides (*mtr*), fumarate (*fccA*), nitrate (*nap*), and DMSO (*dms*), under anaerobic conditions (Charania et al., 2009; Dong et al., 2012). Recently, Kasai et al. (2015) investigated the transcriptional mechanisms for the *mtr* genes (*omcA* and *mtrCAB*), and demonstrated that CRP directly regulates the expression of these genes by binding to the upstream regions of *omcA* and *mtrC* (Figure 3). Several studies have also demonstrated that expression of the *mtr* genes is up-regulated under electron acceptor-limited conditions (Beliaev et al., 2005; Teal et al., 2006; Pirbadian et al., 2014; Kasai et al., 2015), suggesting that MR-1 controls the expression of these genes in response to intracellular redox or energy status. However, the signal-sensing mechanisms underlying the regulation of the *mtr* genes remain unknown, as CRP is not considered to contain redox-sensing domains, such as PAS (Taylor and Zhulin, 1999). As the addition of cAMP to aerobic cultures of MR-1 results in significant induction of fumarate–reductase activity (Saffarini et al., 2003), intracellular cAMP concentration is likely a key determinant of the ability of MR-1 cells to reduce anaerobic electron acceptors. However, intracellular cAMP concentrations in *Shewanella* are unclear.

In addition to CRP, other transcriptional regulators may be directly or indirectly involved in the regulation of the *mtr* genes. Kasai et al. (2015) reported that the expression of *omcA* and *mtrC* is differentially regulated under different culture conditions, although the transcriptional promoters upstream of these genes (P*_omcA_* and P*_mtrC_*; Figure 3) are both dependent on CRP. Interestingly, the researchers also found that deletion of a region upstream of the CRP-binding site of P*_omcA_* resulted in a significant increase in promoter activity under aerobic conditions, suggesting that a yet-unidentified regulator(s) binds to the deleted region and negatively regulates the expression of *omcA* (Kasai et al., 2015). These observations indicate that MR-1 possesses regulatory systems for tuning the composition of OM-cyts in response to changes in environmental conditions, despite that the ratio of MtrC to OmcA has been determined only under limited culture conditions (e.g., 2:1; Shi et al., 2006).

Evidence also suggests that ArcA, Fnr (also referred to as EtrA), and Fur may be involved in the transcriptional regulation of the *mtr* genes in *Shewanella*. ArcA is a DNA-binding response regulator of the bacterial *a*erobic *r*espiration *c*ontrol (Arc) regulatory system and has been well characterized functionally in *E. coli*. The *E. coli* Arc system consists of ArcA and the sensor histidine kinase ArcB, which acts as an indirect oxygen sensor by sensing the redox state of ubiquinone and menaquinone (Georgellis et al., 2001; Malpica et al., 2004; Bekker et al., 2010). Studies have demonstrated that *S. oneidensis* MR-1 has an atypical Arc system consisting of three components, ArcS, HptA, and ArcA, and that the target genes of the MR-1 Arc system substantially differs from those of *E. coli* (Gao et al., 2008; Lassak et al., 2010, 2013). Gao et al. (2008) reported that the expression of several cytochrome *c* genes, including *cymA*, *omcA*, and *mtrC*, is significantly decreased in an *arcA*-deletion mutant of MR-1. Despite this finding, the regulation of the *mtr* genes by ArcA appears to be indirect, as consensus binding sequences for this regulator are not found in the upstream regions of *omcA* and *mtrC* (Gao et al., 2008).

*F*umarate *n*itrate-*r*eduction regulator (Fnr) is another transcriptional regulator that is reported to influence the expression of the *mtr* genes. In *E. coli*, Fnr functions as a sensory protein for environmental oxygen levels by directly reacting with oxygen through a 4Fe–4S cluster (Crack et al., 2004). Cruz-García et al. (2011) reported that the expression levels of *omcA*-*mtrCAB* and *cymA*, as well as other anaerobic respiratory genes, such as *nap*, *fccA*, and *dms*, are decreased in a *fnr*-deletion mutant of MR-1, suggesting that Fnr acts as a global regulator of many anaerobic catabolic processes in MR-1. However, the authors reported that the deletion of *fnr* did not significantly affect the reduction rates of Fe(III) and Mn(IV) oxides, indicating that, unlike CRP, Fnr only plays a minor role in regulation of the Mtr pathway.

Evidence also suggests that *f*erric *u*ptake *r*egulator (Fur) and intracellular iron levels affect expression of the *mtr* genes and the EET activity of *Shewanella* spp. Fur acts as a sensor for intracellular iron levels in many Gram-negative bacteria, including *E. coli* and *Shewanella* (Hantke, 2001; Wan et al., 2004). Fur complexes with ferrous iron (Fe^2+^) and regulates the transcription of many genes, including those related to iron uptake and homeostasis (Griggs and Konisky, 1989; Andrews et al., 2003). Considering that large amounts of iron are required for the synthesis of decaheme OM-cyts, it is reasonable to speculate that expression of the *mtr* genes is responsive to intracellular iron concentrations. In support of this assumption, a previous study reported that *mtr*-gene expression was repressed by iron depletion and induced by iron repletion (Yang et al., 2009). In addition, Kouzuma et al. (2012) also demonstrated that iron uptake supported by siderophore synthesis enhances transcription of the *mtr* genes under Mn(IV)-reducing conditions, suggesting that the intracellular iron concentration is a key determinant of the expression levels of the *mtr* genes. Furthermore, Yang et al. (2013) reported that Fur is involved in the regulation of *mtr* homologs in *S. piezotolerans* WP3 by directly binding to the upstream region of an *omcA* homolog (swp3277). In MR-1, the deletion of the *fur* gene decreases *mtr*-gene expression, and a putative Fur-binding site is located upstream of *omcA* (Wan et al., 2004). However, a subsequent study indicated that regulation of the *mtr* genes in MR-1 is iron responsive but Fur independent, as the transcription of these genes is repressed by iron depletion, even in a *fur*-deletion mutant (Yang et al., 2008). Thus, additional studies are required for elucidating the signal-transduction mechanisms underlying the iron-responsive transcription of the *mtr* genes in MR-1.

The above-mentioned studies indicate that, although the cAMP/CRP-dependent regulatory system plays a direct role in *mtr*-gene regulation, the transcription of these genes is also affected by other regulatory systems. However, it remains to be elucidated how these regulatory systems conjunctively influence the regulation of the Mtr pathway. Gao et al. (2010) have reported in MR-1 that ArcA represses *fnr* and its own transcription and that Fnr also represses *arcA* transcription, indicating that these two regulatory genes are interactively controlled. In addition, the authors have also found that the expression of *crp* is independent of ArcA and Fnr, although it is currently unclear how *crp* is regulated in *Shewanella*. Further investigation is therefore necessary to identify and fully understand the complex environmental-sensing and regulatory networks that regulate the Mtr pathway in MR-1.

## Extracellular EET Components

The synthesis and secretion of electron shuttles, such as riboflavin and FMN, are important for EET by *Shewanella* cells. Previous studies have demonstrated that riboflavin and FMN are secreted at concentrations between 250 nM and 1 μM in cultures of MR-1 and other *Shewanella* strains (Marsili et al., 2008; von Canstein et al., 2008; Coursolle et al., 2010), and it is reasonable to speculate that *Shewanella* possesses specific molecular mechanisms for extracellular secretion of flavins. Covington et al. (2010) isolated a MR-1 mutant with decreased ability to secrete riboflavin and FMN, and found that the disruption of *ushA*, which encodes a putative 5′-nucleotidase, resulted in the accumulation of flavin adenine dinucleotide (FAD) in the culture supernatant, along with decreased levels of FMN and riboflavin. Since UshA was located to the periplasmic space and was shown to catalyze the hydrolysis of FAD to FMN (Covington et al., 2010), MR-1 appears to secrete FAD into the periplasm, where it is then hydrolyzed to FMN by UshA. The synthesized FMN likely diffuses through OM pores into the extracellular space and mediates electron-transfer reactions between OM-cyts and extracellular electron acceptors. Riboflavin appears to be produced by the spontaneous hydrolysis of FMN (Covington et al., 2010), and may also contribute to EET reactions.

Studies on MR-1 have also revealed the involvement of other extracellular components in the transfer of electrons to solid metals and electrodes. For example, Gorby et al. (2006) and El-Naggar et al. (2010) reported that, under O_2_-limited conditions, MR-1 produces conductive pilus-like structures (referred to as nanowires) that appear to be involved in the reduction of solid Fe(III) oxides and electricity generation in MFCs. MtrC and OmcA were also shown to be required for not only the EET activity of MR-1 cells, but also for the conductivity of nanowires (Gorby et al., 2006; El-Naggar et al., 2010). Consistent with these observations, Pirbadian et al. (2014) recently demonstrated that MR-1 nanowires are not pilus-based structures, but rather, extensions of the OM and periplasm that include OM-cyts. Interestingly, these authors also provided evidence suggesting that nanowire filaments are formed from chains of membrane vesicles released from MR-1 cells (Pirbadian et al., 2014).

Cell-surface polysaccharides (CSPs) and other biofilm-related components are also reported to influence the EET activity of MR-1. For example, Kouzuma et al. (2010) reported that a mutant deficient in a CSP biosynthesis gene, SO_3177, generated 1.5-fold higher current than wild-type MR-1 in an MFC. In addition, the SO_3177-deficient mutant (ΔSO_3177) also formed larger colonies with a rough surface and exhibited an enhanced ability to adhere to graphite electrodes. Notably, the surface of ΔSO_3177 cells was more hydrophobic than that of wild-type MR-1 cells, suggesting that cell surface hydrophobicity influences the adhesiveness of MR-1 cells to graphite electrodes and current generation in MFCs (Kouzuma et al., 2010). Altered current generation has also been observed for several transposon- (Tn-) insertion mutants of MR-1, including those with Tn insertion in a putative pilus biosynthesis gene (SO_3350) and a gene with unknown function (SO_4704; Tajima et al., 2011). More recently, Kouzuma et al. (2014) identified a Tn-insertion mutant of MR-1 with distinct colony morphology and high current-generating ability. DNA-microarray analyses of this mutant revealed that a number of genes, including those involved in CPS biosynthesis and biofilm formation, were differentially expressed compared to wild-type MR-1, also suggesting the importance of cell-surface structures for current generation by MR-1.

## Lactate and Pyruvate Metabolism

**FIGURE 4:**
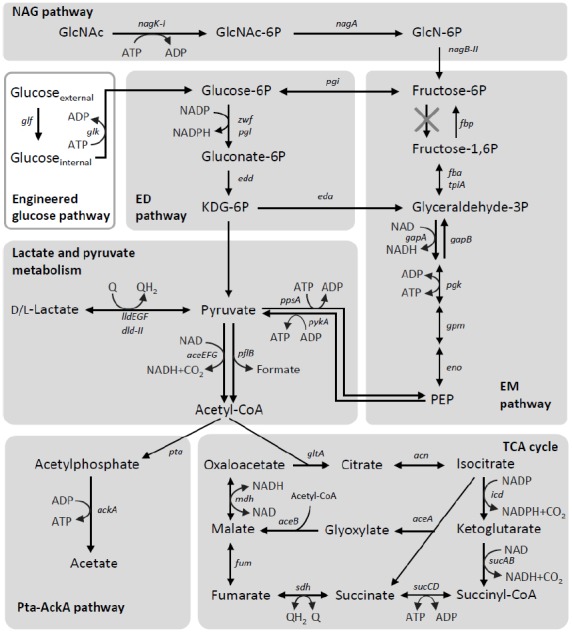
**Carbon-catabolic pathways in *S. oneidensis* MR-1.** The pathways are depicted based on findings reported in the literature (Scott and Nealson, 1994; Yang et al., 2006; Tang et al., 2007a,b; Pinchuk et al., 2009; Choi et al., 2014). Intrinsic catabolic pathways are shown in shaded boxes, while an engineered pathway is depicted in a white box. Q, oxidized form of ubiquinone or menaquinone; QH_2_, reduced form of ubiquinone or menaquinone.

Carbon catabolism is comprised a series of enzymatic reactions, in which reducing equivalents, such as NADH, formate, and reduced quinones, are produced from the oxidation of organic matter. The generated reducing equivalents must be removed from the cell for the catabolic reactions to proceed. In MR-1, these reducing equivalents are utilized for the transfer electrons to extracellular electron acceptors via the EET pathway. As can be seen from the main catabolic pathways in MR-1 (Figure 4), this strain prefers to catabolize low-molecular-weight organic acids, including lactate and pyruvate (Myers and Nealson, 1988a,b; Scott and Nealson, 1994; Serres and Riley, 2006). MR-1 is able to utilize either D- or L-lactate stereoisomers under both aerobic and anaerobic conditions (Pinchuk et al., 2009). In many aerobic bacteria, the oxidation of lactate to pyruvate is catalyzed by membrane-bound respiratory D- and L-lactate dehydrogenases (D- and L-LDH) that use oxidized quinones as electron acceptors (Kohn and Kaback, 1973; Futai and Kimura, 1977; Ma et al., 2007). Although no homologs of previously characterized bacterial respiratory D- and L-LDHs are present in the MR-1 genome or any other sequenced genome of *Shewanella* spp., a study using a comparative genomic approach identified a MR-1 gene cluster (SO_1522 to SO_1588) consisting of a putative lactate permease gene [*lldP* (SO_1522)] and candidate LDH genes for oxidative lactate utilization (Pinchuk et al., 2009). The putative D-LDH gene [*dld-II* (SO_1521)] is a distant homolog of FAD-dependent LDH in yeast, whereas L-LDH is predicted to be comprised three subunits encoded by *lldEGF* (SO_1520 to SO_1518). Genetic and biochemical characterization confirmed that *dld*-II and *lldEFG* encode functional D- and L-LDHs, respectively (Pinchuk et al., 2009). Although these enzymes represent novel types of bacterial LDHs, the results from comparative genomic analysis suggest that homologs of Dld-II and LldEFG are present not only in *Shewanella* and its close relatives, but also in diverse bacteria, including members of *Alphaproteobacteria* and *Betaproteobacteria* (Pinchuk et al., 2009). Notably, although MR-1 utilizes both D- and L-lactate as energy sources, Brutinel and Gralnick (2012) have reported that this strain preferentially utilizes D-lactate, likely due to the inhibition of L-lactate utilization by D-lactate. However, the molecular mechanisms underlying this inhibitory effect remain to be elucidated. These researchers also demonstrated that LlpR (L-*l*actate-*p*ositive regulator, SO_3460) is required for L-lactate utilization by MR-1, although the regulatory mechanisms, including the role of LlpR, in the transcription of LDH genes have not yet been determined.

The end products from lactate metabolism in MR-1 are determined by the growth conditions (Scott and Nealson, 1994; Tang et al., 2007a,b). Under fully aerobic conditions, MR-1 utilizes the complete TCA cycle and therefore does not produce any metabolites from lactate other than CO_2_, because the pyruvate produced from lactate is completely oxidized into CO_2_ (Tang et al., 2007a). In contrast, under anaerobic conditions, including electricity-generating conditions, MR-1 produces acetate as the main metabolite from lactate (Tang et al., 2007a; Lanthier et al., 2008), mainly due to the decreased activity of enzymes involved in acetate oxidation and the TCA cycle (Scott and Nealson, 1994). Under these conditions, MR-1 appears to obtain a substantial portion of ATP by substrate-level phosphorylation in the phosphotransacetylase-acetate kinase (Pta-AckA) pathway (Scott and Nealson, 1994; Tang et al., 2007a; Hunt et al., 2010; Figure 4). In this pathway, pyruvate is oxidized by the pyruvate dehydrogenase (PDH) complex [*aceEFG* (SO_0424 to SO_0426)] and/or pyruvate formate-lyase [*pflB* (SO_2912)], resulting in the formation of acetyl-CoA and CO_2_ or formate, respectively (Pinchuk et al., 2011). The conversion of acetyl-CoA to acetate is catalyzed by acetyltransferase [*pta* (SO_2916)] and acetate kinase [*ackA* (SO_2915)], where ATP is synthesized by substrate-level phosphorylation (Scott and Nealson, 1994; Tang et al., 2007a). As *ack*- and *pta*-deletion mutants of MR-1 are unable to grow on lactate as the sole electron donor and fumarate or Fe(III) citrate as the electron acceptor (Hunt et al., 2010), the Pta-AckA pathway appears to have a crucial role in anaerobic lactate utilization by MR-1.

## Sugar Metabolism

Although glucose is an important source of carbon and energy for diverse heterotrophs, and is often used as a growth substrate in various biotechnology processes, including bioelectrochemical systems (Pham et al., 2003; Logan and Regan, 2006), *S. oneidensis* MR-1 lacks a complete glycolytic pathway and is therefore unable to grow on glucose (Myers and Nealson, 1988a; Serres and Riley, 2006; Rodionov et al., 2010). Of the two main pathways for bacterial glucose catabolism, the Embden–Meyerhof–Parnas (EMP) and Entner–Doudoroff (ED) pathways (Figure 4), the genome of MR-1 has been shown to code for all of the enzymes needed to reconstruct the ED pathway (Rodionov et al., 2010). The EMP pathway of MR-1 is incomplete, as the genome lacks the gene encoding 6-phosphofructokinase, a key enzyme in this pathway (Figure 4; Serres and Riley, 2006; Rodionov et al., 2010), and a glucose/galactose transporter (GluP, SO_2214) is not functional as a result of a frameshift mutation (Romine et al., 2008; Rodionov et al., 2010). In addition, because a glucokinase gene (*glk*) is also not encoded in the genome, MR-1 is unable to catabolize glucose. Although MR-1 has a complete set of genes encoding the phosphoenolpyruvate (PEP):glucose phosphotransferase system (PTS^Glc^; *ptsHI*-*crr* and *ptsG*), it is known that this system does not support growth on glucose via the ED pathway, as this glycolytic pathway cannot produce a sufficient amount of PEP for the phosphotransferase reaction (Rodionov et al., 2010). However, MR-1 is capable of growing on *N*-acetylglucosamine using the NAG and ED pathways under aerobic and electrode-respiring conditions (Figure 4; Yang et al., 2006; Rodionov et al., 2010).

An interesting feature of *S. oneidensis* MR-1 is that spontaneous mutants able to grow on glucose relatively easily arise, after culture media are supplemented with glucose under aerobic conditions. Biffinger et al. (2008, 2009) showed that glucose was utilized for current generation by *S. oneidensis* after a relatively long adaptation period, when oxygen was supplied to MFCs. Howard et al. (2012) reported that, when exposed to glucose under aerobic conditions, MR-1 gained relatively frequently the ability to utilize glucose. Unfortunately, it remains not to be identified how these mutants gained the ability to catabolize glucose. On the other hand, it is also shown that the introduction of glucose facilitator (*glf*) and glucokinase (*glk*) genes of *Zymomonas mobilis* allowed MR-1 to generate current using glucose as the electron donor in MFC (the engineered glucose pathway in Figure 4) (Choi et al., 2014).

## TCA Cycle and its Regulation

The metal-reducing and current-generating bacteria identified to date preferentially utilize low-molecular-weight organic acids, such as lactate and acetate, as carbon and energy sources. As these organic acids are catabolized via the TCA cycle, the metabolic activity of this pathway is an important factor determining the EET activity of current-generating bacteria. *Geobacter* spp. are capable of completely oxidizing acetate to CO_2_ under metal-reducing and current-generating conditions (Lovley and Phillips, 1988; Bond and Lovley, 2003). In contrast, MR-1 does not appear to utilize the complete TCA cycle during anaerobic respiration and current generation, as several key genes involved in the TCA cycle, including those encoding the 2-oxoglutarate dehydrogenase complex (*sucAB*), are not sufficiently expressed under anaerobic conditions (Scott and Nealson, 1994; Beliaev et al., 2005; Tang et al., 2007a). When MR-1 catabolizes one lactate molecule without utilizing the TCA cycle, one NADH and one formate are released through the partial oxidation of lactate to acetyl-CoA (Figure 4). These metabolites correspond to a total of four electrons, which are one third of the electrons released by the complete oxidation of lactate via the TCA cycle. The low Coulombic efficiencies that are observed in lactate-fed *Shewanella* MFCs are likely attributable to this low recovery of electrons. Newton et al. (2009) reported that the Coulombic efficiencies of lactate-fed air-cathode MFCs inoculated with *S. loihica* PV-4 and *S. oneidensis* MR-1 were 26% and 16%, respectively, as calculated based on the total coulombs produced by the complete oxidation of lactate to CO_2_. In addition, substantial amounts of organic acids, predominantly acetate, were accumulated in the electrolyte of both the PV-4 and MR-1 MFCs, suggesting that the TCA cycle is only partially functional in the *Shewanella* MFCs. However, Matsuda et al. (2013) found that the TCA-cycle activity in *S. loihica* PV-4 cells could be modified by changing the electrode potential of the electrochemical cells. Grobbler et al. (2015) also reported the electrode potential-dependent induction of TCA cycle enzymes in electrochemically active biofilms of MR-1. Taken together, these findings suggest that both the extracellular and intracellular redox states are key determinants controlling the TCA-cycle activity in *Shewanella* cells, although the underlying molecular mechanisms remain to be elucidated.

Several studies have indicated that the regulatory systems for the TCA cycle genes of *Shewanella* are distinct from those of *E. coli*. In *E. coli*, many genes involved in the TCA cycle are regulated by the Arc two-component regulatory system (Liu and De Wulf, 2004). Under anaerobic conditions, the kinase activity of sensor kinase ArcB is activated by reduced quinones, and phosphorylated ArcA represses target TCA cycle genes, including those encoding citrate synthase (*gltA*), isocitrate dehydrogenase (*icdA*), succinate dehydrogenase (*sdhABCD*), and malate dehydrogenase (*mdh*, Liu and De Wulf, 2004). However, transcriptome analyses of MR-1 have revealed that these TCA cycle genes are not regulated by the Arc system (Gao et al., 2008). It has also been reported that although a few TCA cycle genes in *E. coli*, including *acnA* and *sdhABCD*, are regulated by Fur and the related small RNA, RyhB (Massé and Gottesman, 2002), the corresponding genes in MR-1 are not under the control of the Fur/RyhB-dependent regulatory system (Yang et al., 2010). Further studies are therefore needed to elucidate the regulatory mechanisms for TCA cycle genes in MR-1.

## Conclusion

MR-1 is an extensively studied model organism for understanding the genetics and biochemistry of bacterial EET and electricity generation in MFCs. Current knowledge on the mechanisms by which bacteria generate electricity in MFCs has largely been obtained from studies performed on MR-1. As described in this article, studies have revealed that many cellular components that are directly and/or indirectly involved in bacterial electricity generation have been identified. However, relatively limited information is available concerning how these components cooperatively work for efficiently generating electricity and conserving energy. As available evidence suggests that the EET pathway is regulated by the level of cAMP, which is an indicator of the cellular energetic states (Charania et al., 2009; Kasai et al., 2015), the EET activity appears to be linked to energy conservation. However, further studies are necessary to determine how cAMP levels are controlled in MR-1 cells. As the intracellular energetic and redox states are two major parameters influencing the global regulation of various cellular activities, future studies addressing how global regulatory systems operate in MR-1 to coordinate catabolic and electron-transfer pathways are needed. As MR-1 is a representative environmental bacterium that thrives in changing environments, such studies are expected to provide useful insights into understanding bacterial lifestyles in the natural environment.

### Conflict of Interest Statement

The authors declare that the research was conducted in the absence of any commercial or financial relationships that could be construed as a potential conflict of interest.
